# National Center Biobank Network

**DOI:** 10.1038/s41439-022-00217-6

**Published:** 2022-11-04

**Authors:** Yosuke Omae, Yu-ichi Goto, Katsushi Tokunaga

**Affiliations:** 1 Central Biobank, National Center Biobank Network (NCBN), Tokyo, Japan; 2 Genome Medical Science Project (Toyama), National Center for Global Health and Medicine (NCGM), Tokyo, Japan; 3 Medical Genome Center, National Center of Neurology and Psychiatry (NCNP), Tokyo, Japan

**Keywords:** Population screening, Pathology, Preclinical research, Disease genetics

## Abstract

There are six national centers (6NCs) for advanced and specialized medicine in Japan that conduct basic and clinical research on major diseases that have a substantial impact on national health. Disease-specific bioresources and information collected by each NC are stored in a separate biobank. The National Center Biobank Network (NCBN) was established in 2011 and coordinates the biobanks and researchers of the 6NCs via an open-access database (Catalogue Database: http://www2.ncbiobank.org/Index_en) as an efficient means of providing registered biological resources and data for use in research communities. The NCBN resources are characterized by their high-quality and rich medical information and are available for life science research and for the development of novel testing methodologies (biomarkers), new treatments, and drugs for future health care in the scope of personalized medicine through a deeper understanding of disease pathogenesis. Here, we explain the activities of the NCBN and the characteristics of the NCBN Catalogue Database.

## Biobanks: Japanese network promotes research collaboration

Japan’s National Center Biobank Network (NCBN) has standardized the collection and storage of high-quality disease-related data and samples, offering collaboration opportunities in basic and applied research. Yosuke Omae and colleagues of the NCBN’s Central Biobank write in a review that these resources could be used in the search for new diagnostic and treatment targets. The NCBN was established in 2011 to coordinate the biobanks of six national centers specializing in cancer and pediatric, geriatric, cardiovascular, psychiatric, neurological, muscular, chronic, and infectious diseases. Its open-access catalogue (http://www2.ncbiobank.org/Index_en) includes filters for specific diseases, basic patient data such as age and sex, tissue type (e.g., plasma, DNA, solid tissue), and medical, family, and lifestyle history. As of 31 March 2022, the NCBN had more than 400,000 registered bioresources and 120,081 registered patients with a wide variety of diseases.

## Introduction

The pathogenesis and pathophysiology of many common chronic diseases (e.g., cancer, cardiovascular disease, dementia) are extremely complex and require a multifaceted and integrated research approach to elucidate and overcome them. For rare and undiagnosed diseases, the development of effective treatments requires a similar research approach that extends from fact-finding and basic research to clinical practice. Human-derived samples (bioresources) have traditionally been collected and stored for research purposes, and their effective utilization has promoted cutting-edge disease research. Following the development of omics analysis technologies in recent years, including genomics, the importance of bioresource banking has been recognized, and many countries in Europe and the United States are competing to establish biobanks^1–3^.

## Establishment and activities of the National Center Biobank Network (NCBN)

**Fig. 1:**
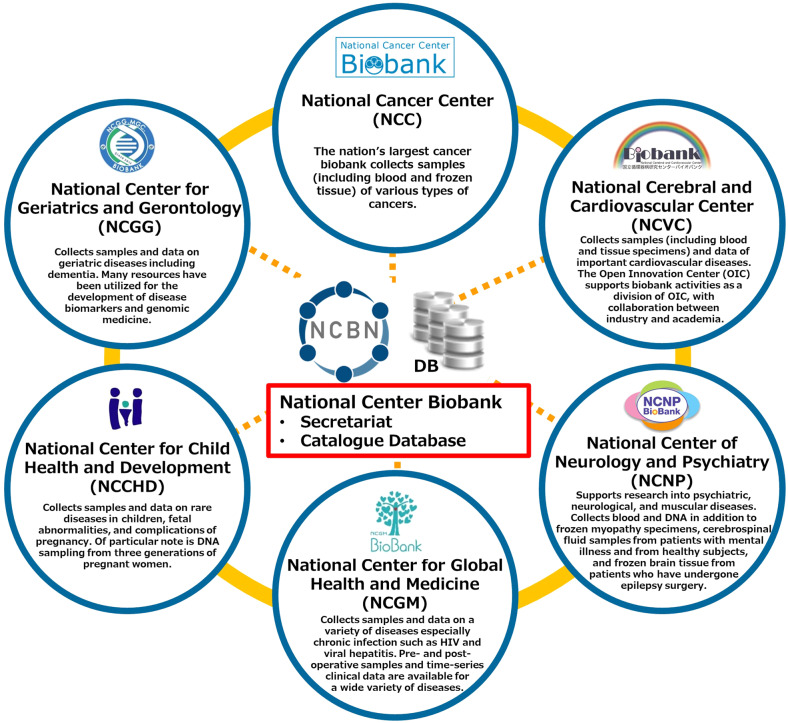
Overview of the NCBN. The NCBN is a collaborative project by six national centers in Japan. National Center Biobank coordinates secretariat and catalogue database to promote medical research and development.

The six national centers (6NCs) for advanced and specialized medicine in Japan are equipped with hospitals and research institutes and conduct basic and clinical research in the following major fields: cancer (National Cancer Center, NCC), cardiovascular diseases (National Cerebral and Cardiovascular Center, NCVC), psychiatric, neurological, and muscular diseases (National Center of Neurology and Psychiatry, NCNP), chronic and infectious diseases (National Center for Global Health and Medicine, NCGM), pediatric diseases (National Center for Child Health and Development, NCCHD), and geriatric diseases (National Center for Geriatrics and Gerontology, NCGG). Each NC has established a disease-based biobank where disease-specific bioresources and information are collected (Table 1). Since the fiscal year 2011, and with the support of the Ministry of Health, Labor and Welfare, the NCBN has been constructing a networked and federated organization of the 6NC biobanks to utilize patient-derived bioresources for medical and research purposes (Fig. 1).

**Table 1:** Overview of 6NC biobanks in NCBN.

NC	Main target diseases	Main types of biological samples	Year of NC establishment	Number of enrolled patients in biobank^1^	Relevant NC URLs
NCC	Cancer	Plasma, DNA, RNA, tumor/non-tumor tissue	1962	54,431	https://www.ncc.go.jp/en/index.html
NCVC	Cardiovascular diseases	Plasma, serum, DNA, pathological tissue, body fluid	1977	22,541	https://www.ncvc.go.jp/english/
NCNP	Psychiatric, Neurological diseases	Plasma, serum, DNA, cerebrospinal fluid, brain tissue, muscle tissue	1986	19,764	https://www.ncnp.go.jp/en/
NCGM	Chronic and Infectious diseases	Plasma, serum, DNA, pathological tissue	1993	20,126	https://www.ncgm.go.jp/en/index.html
NCCHD	Pediatric diseases	Serum, DNA, umbilical cord, placenta, chorionic plate	2002	2,468	http://www.ncchd.go.jp/en/index.html
NCGG	Geriatric diseases	Plasma, serum, DNA, cerebrospinal fluid, urine, feces	2004	12,161	https://www.ncgg.go.jp/english/index.html

^1^As of 31 March 2022.

The Secretariat of the NCBN was established within the NCGM for the purpose of coordinating its implementation. Its activities include (1) standardization of ethical review (comprehensive informed consent acquisition), clinical information (common medical interview questionnaires and disease name registration), and sample handling (standardization and publication of standard operating procedures, SOPs) among the 6NC biobanks; (2) release of the catalogue database; and (3) public relations activities. The first 5-year phase was completed in FY2016, and the second phase began in FY2017, with continuing activities related to clinical information, bioresources, and ethical, legal, and social issues to build a new common platform, as well as activities to further develop the features of the NCBN and promote collaboration with other biobank organizations.

In FY2017, the NCBN established joint meetings with the Research and Development Committee of the Japan Pharmaceutical Manufacturers Association (JPMA), and in FY2020, the GAPFREE project was started with the support of the Japan Agency for Medical Research and Development (AMED) to develop a disease-specific information database integrating multiomics data and clinical information. Since FY2018, the NCBN has participated in the “biobank cross-search system” through the Biobank-Construction and Utilization biobank for genomic medicine Realization (B-Cure)^4^). This collaborative research and development project is led by AMED and promotes the utilization of bioresources among the three major biobanks in Japan: the NCBN, BioBank Japan (BBJ), and Tohoku Medical Megabank Project (ToMMo)^5,6^.

## Characteristics of NCBN bioresources

### High-quality and rich medical information

The primary characteristic of the NCBN is that it includes a variety of patient-derived bioresources, including detailed medical information, which has been collected by the physicians of each specialist NC. In addition to the collection of basic medical information through linkage to electronic medical records, disease-specific clinical information is also available in each NC biobank. For example, the disease severity of each psychiatric disease patient is assessed by the clinical psychologist at the NCNP. Brain imaging test records, such as MRI and PET, are collected at the NCNP and NCGG, and cardiovascular imaging test records, such as electrocardiograms and echocardiograms, are collected at the NCVC. These data are available as accompanying medical information in the NCBN. These patient-derived bioresources and information provide high-quality research resources that can serve as the basis for basic research as well as advanced medical research in drug discovery, personalized medicine, and the development of regenerative medicine^7–12^. This characteristic is based on the physical proximity of the medical and research sites at each NC, which enables the selection of optimal sample collection methods and the gathering of necessary clinical information from the perspectives of both physicians and researchers with sound knowledge of both the disease and the research. An important difference between NC researchers and researchers at universities and other institutions who are studying a particular disease is that NCs are continuously conducting research on the diseases in question, and bioresources are accumulated over a long span of 10 to 20 years. In fact, the NCNP has collected more than 16,000 frozen muscle tissue samples since 1978. This continuity is an indispensable advantage of the NCBN when collecting and using bioresources for rare diseases, and it is necessary for the NCBN to maintain a management policy that does not undermine this advantage.

### Types of bioresources

An overview of the bioresources that can be provided by the NCBN is shown in Table 2. In general, the target of genomic analysis is peripheral blood DNA. However, it is often necessary to confirm the expression and protein levels to assign significance to the identified genomic mutations. In addition to omics analysis, including genomic analysis, biochemical and pathological analyses are also required in life science research. The NCBN intends to create a system that can appropriately process, store, and utilize a wide variety of bioresources for such purposes. This wide range of uses is a characteristic feature of NCBN bioresources.

**Table 2:** Overview of bioresources in the NCBN catalogue database.

NC	Total number	Bioresources						
		Plasma	Serum	DNA^1^	RNA	Cerebrospinal fluid	Solid tissue	Others^2^
6NC	414,046	99,832	77,699	128,491	44,169	4898	34,161	24,796
NCC	154,330	44,169	0	44,169	44,169	0	21,823	0
NCVC	119,386	25,347	26,295	43,216	0	0	531	23,997
NCNP	44,592	7474	5951	16,139	0	4511	10,517	0
NCGM	55,229	11,183	31,691	12,140	0	0	0	215
NCCHD	3239	0	1040	1450	0	0	749	0
NCGG	37,270	11,659	12,722	11,377	0	387	541	584

^1^Including pre-extraction.

^2^Urine, feces, body fluid, etc.

### Genomic information

In addition to the bioresource and clinical information characteristics described above, whole genome sequencing analysis for 9,850 NCBN samples has been conducted since FY2020 with support from AMED. These can be utilized as control subjects in research on cancer and rare diseases in the Japanese population, and their variant frequency information will be registered to the NBDC Human Database and Medical Genomics Japan Variant Database (MGeND) after publication^13,14^. It is now possible to search the catalogue database for bioresources with genomic information.

## Open-access bioresource search of the NCBN Catalogue Database

### Overview of information registered in the catalogue database

**Fig. 2:**
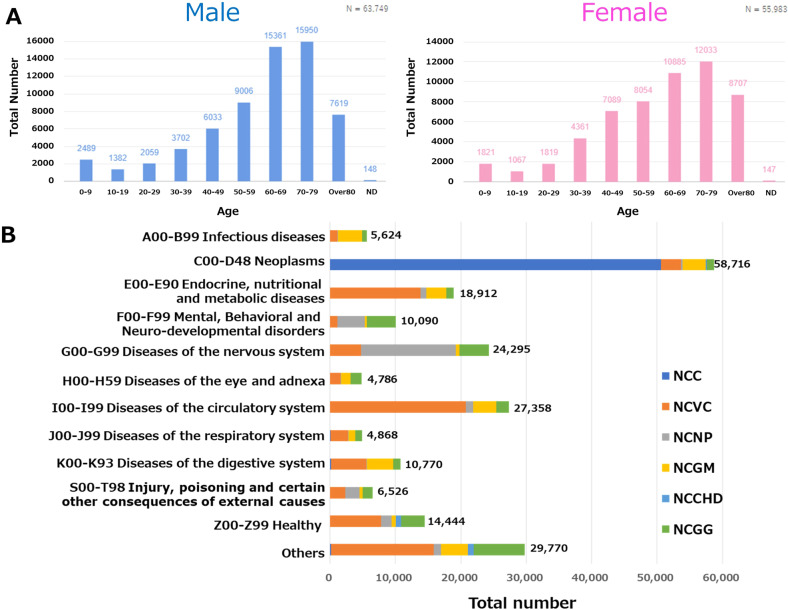
Overview of registered people in the NCBN Catalogue Database [as of March 2022]. **A** Distribution of each sex and age classification. **B** Distribution of each disease classification.

An overview of the catalogue data is shown in Fig. 2A. In addition to basic patient information such as age and sex, other common interview items include information on medical history, family history, allergy history, history of drinking and smoking, and the genome (Table 3). The disease information for primary diseases and comorbidities is registered as the ICD10 code or corresponding MEDIS number. Bioresource information includes the type of specimen (e.g., plasma/serum, DNA, solid tissue, spinal fluid), date of collection, the amount obtained, and method of storage. For pathological specimens, the database also includes SOPs of the storage methods for various types of specimens (https://www.ncbiobank.org/sample/index.php). On 31 March 2022, the total number of registered bioresources exceeded 400,000, and the total number of registered patients was 120,081, with a wide distribution of diseases (Table 2, Fig. 2B). These statistics are updated regularly, and the latest figures can be found in the NCBN Catalogue Database (http://www2.ncbiobank.org/Index_en).

**Table 3:** Overview of information in the NCBN catalogue database.

Basic patient information	Date of birth, sex, height, weight, blood pressure
Medical interview information (medical history)	Cancer, hypertension, diabetes, stroke, heart disease, kidney disease, liver disease, mental disease, current condition
Medical interview information (life history)	Family history, drinking history, smoking history, allergy history, surgical history, blood transfusion history
Disease information	ICD10 code of primary disease and comorbidities, or corresponding MEDIS number
Biological sample information	Type, date of collection, method of storage

### How to use the open-access NCBN Catalogue Database (Fig. 3)

When one accesses the NCBN Catalogue Database via the URL (http://www2.ncbiobank.org/Search/Search_en), a search condition window appears. One can search for a specific disease by name and further narrow the search by including basic patient information (sex, age), type of bioresource, current and previous medical history, family history, smoking or drinking history, and availability of genomic information. In the search by bioresource type, it is also possible to search for the number of samples that can be utilized in companies and academia through sample distribution.

**Fig. 3:**
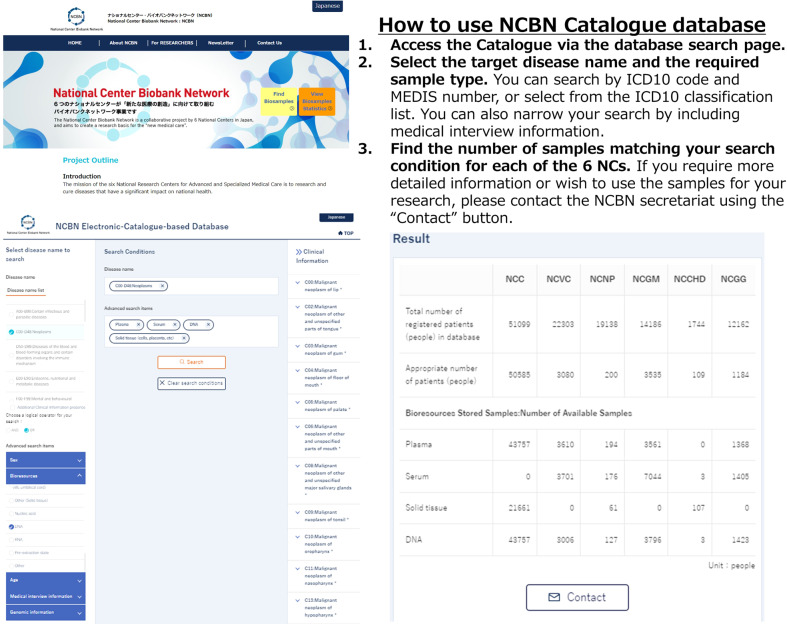
How to use the NCBN Catalogue Database. The bioresource information users need can be searched via the “Search Conditions” page in the open-access NCBN catalogue database. The NCBN secretariat can be contacted using the “Contact” button in the “Result” page.

## Provision of NCBN bioresources

### Contacting the NCBN secretariat

Inquiries from outside parties can be made by e-mail to the central biobank or to individual NC biobanks to obtain information regarding the availability of samples. The 6NCs cover a wide range of diseases, and the types of samples and storage methods are diverse. Some NCs may have bioresources for which detailed data are not available in our catalogue database. If the catalogue database shows that samples matching the inquiry exist at more than one NC, or if more detailed information is required, the NCBN secretariat can be contacted using the “Contact” button. The referring NCBN Catalogue DB search result ID should be provided as the reference (https://www.ncbiobank.org/en/contactus.php).

The NCBN establishes a common platform among the 6NCs for comprehensive informed consent acquisition, enabling future genomic analysis and utilization for research development by companies as the result of our standardization of ethical review. There are two types of provision of bioresources for research use: collaborative research and distribution. Collaborative research involves clinicians and researchers within each NC as coresearchers and is appropriate in the situation when a wealth of clinical information is needed, when the recipient needs to collect the desired medical information anew, or when clinicians and researchers familiar with the characteristics of the disease itself are needed. In contrast, distribution is suitable for research that can be conducted with limited ancillary information and can be made into a Material Transfer Agreement (MTA) in which the provider biobank does not claim intellectual property rights through consultation. At present, only four NCs (NCNP, NCGM, NCCHD, and NCGG) can offer distribution services, but we are aiming to make a distribution available to all NCs and simplify and unify the procedures as much as possible.

### Experience in sample distribution and collaborative research

The summary status of the utilization and publications of each NC biobank is summarized in Table 4. This status is also reported in the NCBN annual report, which is accessible through the NCBN website, and data from the provider institutions are also posted on the website for each NC biobank^15–20^. The “Research activities and achievements” section of the website (https://ncbiobank.org/research/index.php) lists the number of collaborative research projects conducted using NCBN samples and information, the number of collaborative research institutions such as companies and academia, and information about results published since 2010. These are all listed by NC and by year.

**Table 4:** Summary status of utilization of 6NC biobanks.

NC	Total number of collaborative studies	Total number of collaborative institutions			Total number of provision^1^		Total number of relevant publications
		Company	Academia	Other	Collaborative research	Distribution	
NCC	294	237	308	117	39,208 (827)	0 (0)	933
NCVC	61	13	95	28	13,493 (119)	56 (8)^2^	51
NCNP	104	26	107	84	11,036 (110)	2,108 (59)	310
NCGM	126	21	156	74	20,603 (31)	914 (27)	150
NCCHD	82	0	90	6	2185 (10)	352 (7)	172
NCGG	121	44	237	147	58,921 (145)	1,917 (15)	183

^1^Total number of provision bioresources (times).

^2^Exceptional distribution for educational purposes.

## Conclusion

In this review, we provide an overview of the NCBN and NCBN Catalogue Database. The six NCs have continuously collected a wide variety of patient-derived bioresources, including detailed medical information for the diseases in question over a long span of 10 to 20 years, and the NCBN constructs a networked and federated organization of the 6NC biobanks to utilize patient-derived bioresources for medical and research purposes. The NCBN Catalogue Database is an open-access database for the efficient provision of registered biological resources and data for use in research communities. Recently, the total number of obtained genome-wide data for NCBN bioresources has increased for many diseases through whole genome sequencing analysis or SNP array analysis, and the NCBN is creating a system that can provide both bioresources and genomic data to users. Quality control is also important to scientifically support the research use of various sample types. In this sense, it is essential to standardize the hardware and software aspects of registering information in terms of sample collection methods, processing processes, storage conditions, and storage periods. The global standardization of biobanking is steadily progressing, and the NCBN is preparing for ISO20387:2018 biobanking certification^21^.
